# 764 Comparison of Topical Silver Sulfadiazine and Triple Antibiotic Ointment in a Porcine PT Burn Model

**DOI:** 10.1093/jbcr/irad045.239

**Published:** 2023-05-15

**Authors:** Samara Sober, Stacey Wong, Steven Sandoval, Adam Singer

**Affiliations:** Stony Brook Medicine, Stony Brook, New York; Stony Brook Medicine, Stony Brook, New York

## Abstract

**Introduction:**

The standard of care for partial thickness burns is topical antibiotics or occlusive dressing. Silver Sulfadiazine (SSD) emerged at a time of burn wound infection and sepsis prevalence and is now the most used topical worldwide. Despite its antimicrobial benefits, SSD has been shown to inhibit keratinocytes and there is concern that it might delay wound healing. This study compared time to complete wound closure and reepithelialization in partial thickness porcine burns treated with SSD, triple antibiotics ointment or petrolatum ointment

**Methods:**

We created 21 deep partial thickness (DPT) burns per animal on the backs and flanks of 3 anesthetized domesticated pigs (30-35 kg) using an established vertical progression model (aluminum bar preheated to 80° C for 20s). In each pig, burns were randomly assigned SSD, triple antibiotic ointment, or petrolatum ointment (in a 1:1:1 ratio) applied daily for two weeks followed by twice weekly for another 2 weeks. Healing progression was measured by wound digital photography and punch biopsies to assess gross wound closure, reepithelization and scar depth respectively.

**Results:**

The median (IQR) days to complete wound closure were 17 (17-21), 17 (14-21), and 21 (17-21) for wounds treated with SSD, petrolatum and triple antibiotic ointment respectively (p=0.06). The mean percentage of wound reepithelization at days 10, 14, 17, 21, and 28 for wounds treated with, SSD, petrolatum, and triple antibiotic ointment were 10%±20 vs. 8%±14 vs. 3%±12 (p=0.34), 45%±49 vs. 37%±44 vs. 48%±42 (p=0.74), 82%±38 vs. 86%±20 vs. 90%±27 (p=0.7), 74%±38 vs. 65%±50 vs. 93%±16 (p=0.049), and 92%±17 vs. 91%±28 vs. 98%±5 (p=0.4). The mean scar depth was 3.9 (1.1), 4.2 (0.9), and 3.4 (0.9) mm for wounds treated with SSD, petrolatum, and triple antibiotic ointment respectively (p=0.07). RMANOVA analysis showed significant change over time (p< 0.001) and a near significant difference between groups (p=0.054). On macroscopic analysis, appreciable pseudoeschar formation and erythematous allergic reactions were identified on wounds treated with SSD and triple antibiotic ointment respectively.

**Conclusions:**

No significant differences in wound healing were found between groups in this porcine DPT burn model. SSD did not delay closure. Hypersensitivity to topical triple antibiotics was common.

**Applicability of Research to Practice:**

This study provides insight into the topical ointment standard of care for burns and helps establish the standard comparator topical treatment for future preclinical and clinical studies.

